# Mechanistic Analysis of Fluid Dynamics and Multifactorial Impact Mechanisms in Inhaled Pharmaceutical Deposition for Chronic Respiratory Diseases

**DOI:** 10.3390/bioengineering12060643

**Published:** 2025-06-12

**Authors:** Fuli Hu, Songhua Ma, Tianliang Hu

**Affiliations:** Key Laboratory of High Efficiency and Clean Mechanical Manufacture, School of Mechanical Engineering, Shandong University, Jinan 250061, China; 202314323@mail.sdu.edu.cn (F.H.); tlhu@sdu.edu.cn (T.H.)

**Keywords:** respiratory modeling, drug delivery, multiphase flow, aerosol dynamics, computational fluid dynamics, inhaled medication, breath-holding

## Abstract

The clinical efficacy of inhalation therapy in chronic respiratory diseases is fundamentally constrained by particle deposition patterns. This study employs computational fluid dynamics (CFD) and response surface methodology (RSM) to elucidate the mechanistic interplay of deposition determinants through multifactorial sensitivity mapping. The study comprises two key components: (i) the development of an accurate three-dimensional respiratory airway model spanning from the oral cavity to the fifth-generation bronchi and (ii) the integration of a Box–Behnken Design (BBD) experimental framework with computational fluid dynamics simulations. Furthermore, we developed a multifactorial regression model to analyze the synergistic interactions among deposition determinants. The study demonstrated a positive correlation between breath-holding time and drug deposition efficiency, revealing a hierarchical order of critical parameters: peak flow rate > breath-holding time > particle diameter. These findings have important implications for optimizing respiratory drug delivery strategies in clinical settings.

## 1. Introduction

With the intensification of air pollution and the aging of the population, the number of individuals suffering from respiratory diseases such as asthma, emphysema, and tracheitis has been increasing [1]. This vast patient group has led to a growing demand for respiratory medications.

Traditional treatment methods for respiratory diseases often suffer from issues such as low rates of drug utilization, significant side effects, and drug resistance, which compromise clinical efficacy. With the development of medical technology, inhalation drug delivery has emerged as a core approach [2,3,4] in the treatment of pulmonary diseases such as asthma, chronic obstructive pulmonary disease (COPD), and cystic fibrosis; its efficacy critically depends on the deposition patterns of drug particles within the targeted regions of the respiratory tract. Consequently, understanding the interplay between airflow dynamics and particle deposition mechanisms is essential for optimizing drug delivery systems.

To achieve precise drug application, the deposition of particles within the respiratory tract can be explored through experiments and numerical simulations. Experimental techniques for studying the human respiratory tract have been developed, but they are associated with long duration, high costs, and individual differences [5,6]. On the contrary, numerical simulation supports the digitization of respiratory tract models and simulates the deposition of drug particles under various respiratory states, and the distribution of the fluid field and particle deposition can be observed clearly at a very low cost [7].

In recent years, numerical models, notably computational fluid dynamics (CFD), have been extensively employed to simulate respiratory airflow and particle transport processes [8,9,10,11,12]. The physiological structure and functional characteristics of the respiratory tract are dominant determinants of drug delivery patterns. In 1963, Weibel [13] created the earliest respiratory tract model. He simplified the complex structure of the respiratory tract by describing it as a symmetrical bifurcating network. However, this simplified model was unable to predict the true patterns of particle deposition. In 1968, to make the model closer to the real respiratory tract, Horsfield and Cumming [14] built an asymmetrical respiratory tract model based on the Weibel model. This model aligns more closely with actual anatomical models. With the development of medical imaging such as computed tomography (CT) and magnetic resonance imaging, more precise models of the respiratory tract can be constructed [15,16]. These models serve as the foundation for accurate simulations of drug particle deposition within the respiratory tract.

Although significant advancements have been made in respiratory tract modeling, studies indicate that particle characteristics, such as size and density, are equally critical in determining particle deposition outcomes. Cheng et al. [17] explored the deposition patterns of respiratory dust within the respiratory tract. Their data indicated that 1 μm dust particles are more prone to entering the lungs, whereas 80 μm dust particles tend to accumulate within the nasal cavity. Ciloglu and Karaman [18] investigated the deposition patterns of particles within the respiratory tract of healthy individuals. Their research revealed that the deposition efficiency increases as the particle diameter increases. Zhang et al. [19] investigated the influence mechanisms of both particle diameter and density within the respiratory tract. The study found that at the same inhalation flow rate, the overall deposition fraction of drug particles gradually increased with particle diameter and density. Guo et al. [20] investigated the impact of particle density on deposition patterns and demonstrated that compared to inhalation flow rate and particle diameter, density plays a secondary role in influencing deposition efficiency. Notably, the aforementioned studies on particle characteristics have been conducted under the assumption of constant airflow velocity, whereas research on particle behavior under dynamic airflow variations during the respiratory process still requires further exploration.

Furthermore, in clinical practice, patients are often advised to perform breath-holding maneuvers following the inhalation of medication to prolong the residence time of particles in the deep lung regions [21]. Despite the established clinical relevance of this maneuver [22], the specific effects of breath-holding time on transient airflow behavior within inhalations and particle deposition redistribution remain quantitatively understudied. Existing literature has predominantly focused on simulating steady-state inhalation phases [23,24], neglecting the transient dynamics of post-inhalation breath-holding—a period during which airflow stagnation and prolonged particle residence time may significantly alter deposition efficiency through mechanisms such as secondary flow or enhanced diffusion.

**Figure 1:**
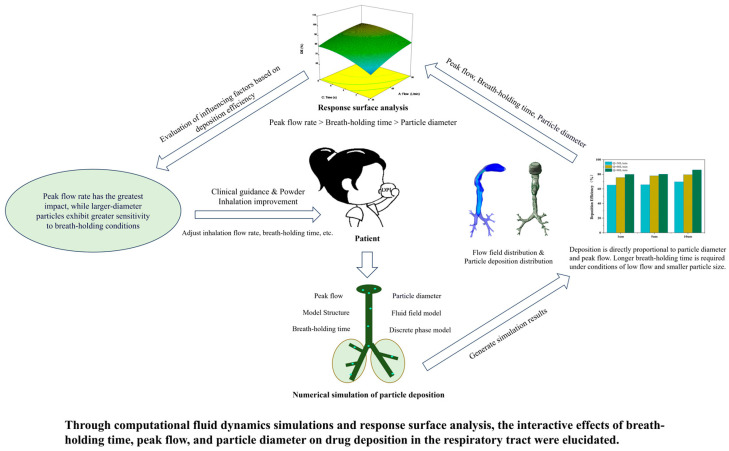
Research procedure and methodology diagram.

Based on a physiologically realistic human respiratory tract model, this study aims to evaluate the impact factors, especially breath-holding, on drug particle deposition. The procedure of the research is shown in Figure 1. After employing the Box–Behnken design (BBD) to systematically deploy the simulation scheme across various breath-holding times (0–8 s), particle diameters (1–10 μm), and respiratory patterns (with peak flow rates ranging from 30 to 90 L/min), the transient airflow fields and particle deposition can be simulated with the help of the combination of CFD and the discrete phase model (DPM). Response Surface Methodology (RSM) was preferred to establish a regression model, which quantifies the relative significance of breath-holding time, particle diameter, and peak flow rate on the regional particle deposition. It can be concluded that the peak flow rate has the most pronounced impact on respiratory particle deposition, followed by breath-holding duration and particle diameter. This study provides an alternative numerical method for the design verification of the practical drug inhaler.

## 2. Materials and Methods

### 2.1. Establish a 3D Model of the Human Respiratory Tract

**Figure 2:**
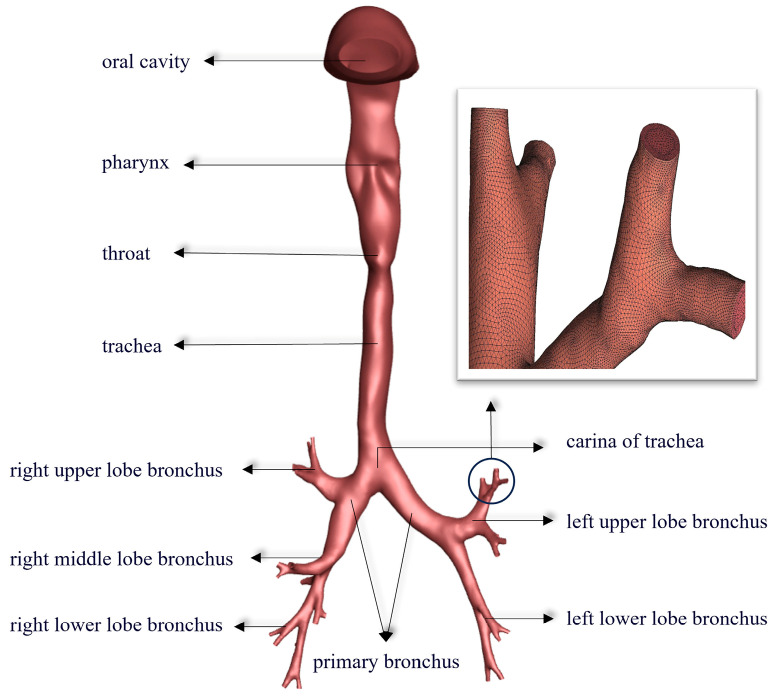
Three-dimensional respiratory tract model based on CT imaging with structural details.

With the support of the Respiratory Department of Qilu Hospital (Jinan, China), a 3D model of the respiratory tract was established using MIMICS^®^ v21.0 (Materialise, 2020, Leuven, Belgium) software based on the CT images of a healthy adult male’s lungs. This model comprises the oral cavity, pharynx, larynx, and trachea and extends down to the fifth-level bronchus (Figure 2).

**Figure 3:**
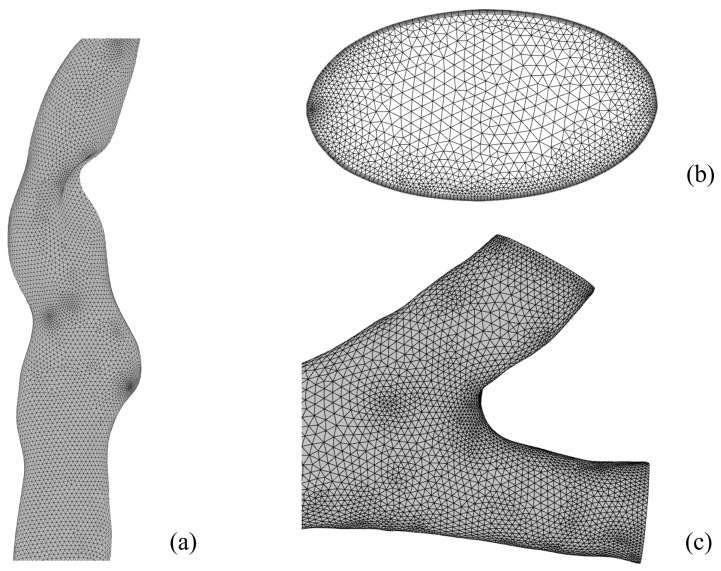
Various views of the generated mesh. (**a**) Pharynx and larynx; (**b**) inlet; (**c**) bronchus.

Considering the irregularity of the respiratory tract, the model was meshed using an unstructured tetrahedral mesh. Compared to a structured mesh, an unstructured mesh is suitable for complex geometric structures and computational domains with variable flow fields. The mesh is refined at the tracheal bifurcation and in the minute bronchioles to accurately capture the flow conditions of the air current along the wall. Meanwhile, to enhance simulation accuracy, 5 layers of prism mesh are employed near the wall, with the first layer having a height of 0.05 mm and the mesh growth rate of each subsequent layer being 1.18. The different views of the generated mesh are depicted in Figure 3.

**Figure 4:**
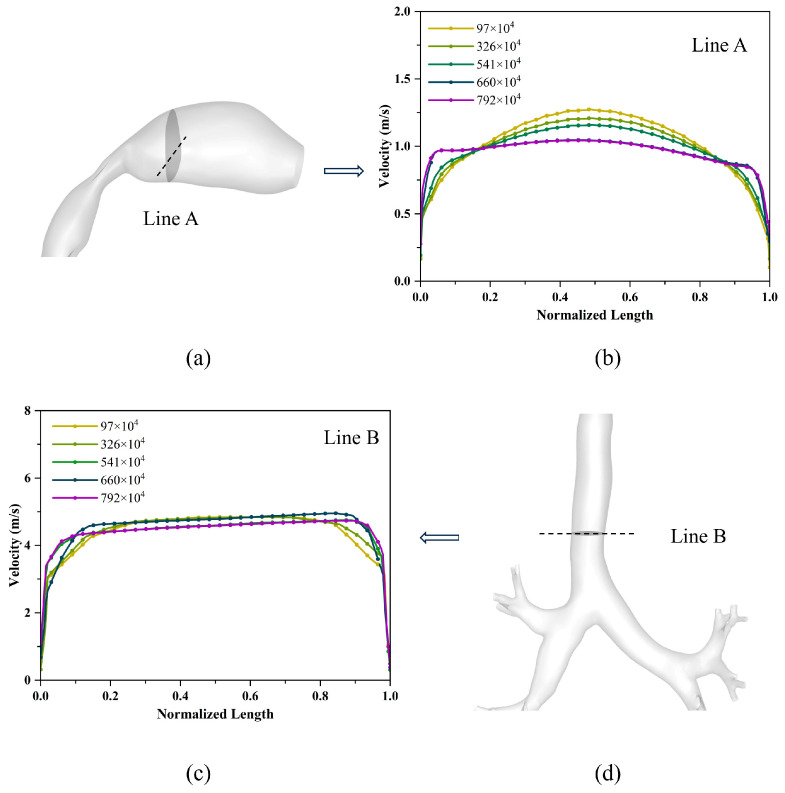
Verification of mesh independence. (**a**) Position of line A used for velocity profiles; (**b**) velocity profiles for different mesh numbers on line A; (**c**) velocity profiles for different mesh numbers on line B; (**d**) position of line B used for velocity profiles.

The density and precision of the mesh can affect simulation results. To ensure the reliability and accuracy of the simulation results, models with mesh counts of 970,000, 3,260,000, 5,410,000, 6,600,000, and 7,920,000 were selected. Transient simulations were conducted under the conditions of an inlet flow rate of 60 L/min. The results are shown in Figure 4, which displays the normalized velocity curves along the axis of two cross-sections for models with different mesh counts. There was no significant difference in the axial velocity of the cross-section between the models with mesh counts of 6,600,000 and 7,920,000. Based on comprehensive consideration of the accuracy of numerical simulation and time cost, the model with 6,600,000 mesh counts was preferred for the study on particle deposition in the respiratory tract.

### 2.2. Boundary Conditions

The meshed model was imported into ANSYS^®^ 2021 R1 (ANSYS Inc., 2021, Canonsburg, PA, USA) Fluent software for simulation under non-steady-state conditions. The actual respiratory process is highly intricate, making it challenging to fully encompass all its characteristics in numerical simulations. Thus, reasonable assumptions about the respiratory process are made during the simulation process. In this paper, we employ a prevalent simplification approach by idealizing the respiratory flow rate as a sine function [10,25].

**Figure 5:**
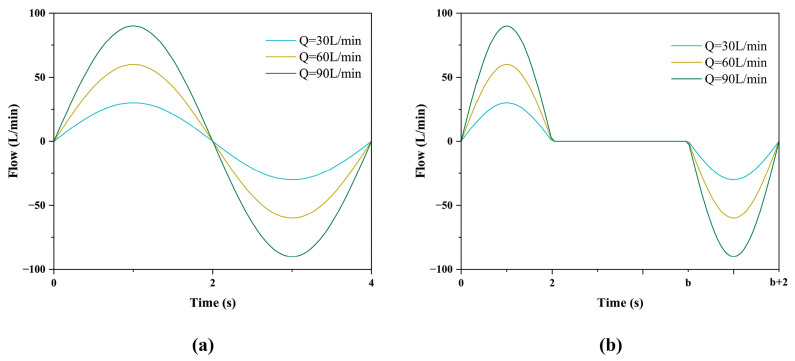
Respiratory waveform. (**a**) Different levels of cyclic respiratory waveforms, (**b**) waveforms of different levels of cyclic breathing plus breath holding.

Inlet boundary condition: We included three respiratory conditions to precisely simulate respiratory states under different scenarios, with peak flow rates of 30, 60, and 90 L/min (Figure 5a)

These flow rates correspond to different levels of breathing effort exerted by patients during the administration of inhaled medications. The general respiratory frequency for adults is 15–20 breaths/min. Thus, the respiratory cycle is set to 4 s. The oral cavity’s inlet is set as a velocity inlet, with the direction being perpendicular to the normal direction of the inlet plane. The velocity formula at the inlet can be given as:

(1) $$\begin{array}{c} y=\frac{Q}{A}\sin\left(\frac{2\pi }{T}t\right) \end{array}$$

During breath-holding, there is no gas exchange between the respiratory tract and the external atmosphere, so the inlet flow is set to 0 L/min at this time. The respiratory flow rate for exhalation and inhalation follows the sinusoidal function under cyclic breathing conditions (Figure 5b), the inlet velocity formula during breath-holding is set as:

If 0 ≤ *t* ≤ 2, we have:

(2) $$\begin{array}{c} y=\frac{Q}{A}\sin\left(\frac{2\pi }{T}t\right) \end{array}$$

If 2 ≤ *t* ≤ *b*, we have:

(3) $$\begin{array}{c} y=0 \end{array}$$

If b ≤ *t* ≤ (*b* + 2), we have:

(4) $$\begin{array}{c} y=\frac{Q}{A}\sin\left(\frac{2\pi \left(b-t\right)}{T}\right) \end{array}$$

where y represents the inlet velocity, *Q* is the respiratory peak flow rate, *A* is the area of the velocity inlet, and *b* takes values of 4, 5, 6, 8, 10, and 11, representing breath-holding times of 2, 3, 4, 6, 8, and 9 s, respectively. *t* represents the temporary time, and *T* represents one complete respiratory cycle.

Outlet boundary condition: The terminus of the bronchus is set as a pressure outlet. The discrete phase type is selected as “escape,” indicating that particles escaping from the outlet will not be tracked further and are assumed to be fully captured by the deeper bronchial wall.

Wall condition: The wall is set as a no-slip wall with a standard roughness model. The thermal effect generated by fluid-wall friction is not considered. The discrete phase type is selected as “trap,” indicating that particles are “captured” by the wall at a certain distance from it, and the position information at the time of capture is recorded.

### 2.3. Preferred Simulation Model

#### 2.3.1. General Fluid Field Model

In CFD, the motion of fluids is governed by three fundamental equations: the law of conservation of mass; the law of conservation of momentum; and the law of conservation of energy. These three laws are represented by the Navier–Stokes (N–S) equations (which encompass the continuity equation), the momentum equation, and the energy equation. In the present study, energy exchange was not considered. Thus, only the continuity equation and momentum equation were employed. Due to the irregular shape of the respiratory tract, turbulent flow exists in the airflow within it. The shear stress transport (SST *k-ω*) model is a turbulence model based on the RANS equations. This model combines the advantages of the *k-ω* model (a turbulence model based on turbulent kinetic energy (*k*) and specific dissipation rate (*ω*)) and the *k-ε* model (a turbulence model based on turbulent kinetic energy (*k*) and turbulent kinetic energy dissipation rate (*ε*)) and can accurately simulate complex flow states such as boundary layer flows and free shear flows, providing precise turbulent boundary conditions [26]. Among them, the main equations describing the airflow are shown as follows.

Continuity equation:

(5) $$\begin{array}{c} \frac{\partial \rho }{\partial t}+\nabla \cdot \left(\rho u_{i}\right)=0 \end{array}$$

where ρ represents the density of the fluid, t is time, $u_{i}$ is the velocity vector of the fluid in the i-direction, and ∇ is the gradient operator.

Momentum equation:

(6) $$\begin{array}{c} \frac{\partial \left(\rho \mu_{i}\right)}{\partial t}+\frac{\partial \left(\rho \mu_{i}\mu_{j}\right)}{\partial x_{j}}=-\frac{\partial p}{\partial x_{j}}+\frac{\partial \tau_{ij}}{\partial x_{j}}+\rho g_{i}+F_{i} \end{array}$$

where *p* represents the static pressure, $\tau_{ij}$ represents the viscous stress tensor, $g_{i}$ represents the gravitational force in the i-direction, and $F_{i}$ represents other forces acting on the fluid.

Turbulent kinetic energy k:

(7) $$\begin{array}{c} \frac{\partial \left(\rho k\right)}{\partial t}+\frac{\partial \left(\rho ku_{i}\right)}{\partial x_{i}}=\frac{\partial }{\partial x_{j}}\left(\Gamma_{k}\frac{\partial k}{\partial x_{j}}\right)+G_{k}-Y_{k}+S_{k} \end{array}$$

Specific dissipation rate ω:

(8) $$\begin{array}{c} \frac{\partial \left(\rho \omega \right)}{\partial t}+\frac{\partial \left(\rho \omega u_{i}\right)}{\partial x_{i}}=\frac{\partial }{\partial x_{j}}\left(\Gamma_{\omega }\frac{\partial \omega }{\partial x_{j}}\right)+G_{\omega }-Y_{\omega }+S_{\omega } \end{array}$$

where $\Gamma_{k}$ and $\Gamma_{\omega }$ are the effective diffusion coefficients for k and ω, respectively. $G_{k}$ and $G_{\omega }$ represent the production of turbulent kinetic energy and turbulent dissipation rate due to the mean velocity gradient, respectively. $Y_{k}$ and $Y_{\omega }$ denote the dissipation of k and ω due to turbulence, respectively, and $S_{k}$ and $S_{\omega }$ represent custom source terms.

#### 2.3.2. Discrete Phase Model

For particles flowing in a fluid, the Euler–Lagrange method is employed for simulation. The DPM describes particle motion based on the Lagrangian approach, which is capable of tracking the trajectories of individual particles within the fluid. The DPM disregards the volume of particles and considers the interaction between particles and the fluid but neglects interparticle forces. In the present study, the particle model was treated as spherical. Due to the significantly higher density of the discrete phase compared with the continuous phase, forces such as the Basset force, virtual mass force, and pressure gradient force were neglected. Heat- and mass-transfer processes were not considered, so the thermophoresis force was also ignored. Given that the particle diameters of the inhaled medication ranged from 1 to 10 μm, and the particle diameters selected in this study are 1, 5, and 10 μm; the Saffman force and Brownian force, which are relevant only at the nanoscale, were disregarded. Therefore, the primary forces considered were drag force and buoyancy force.

The force balance equation for particles can be expressed as:

(9) $$\begin{array}{c} m_{p}\frac{du_{pi}}{dt}=F_{i}={\overset{⃑}{F}}_{D}+{\overset{⃑}{F}}_{R} \end{array}$$

where ${\overset{⃑}{F}}_{D}$ represents the drag force, and its expression is:

(10) $$\begin{array}{c} {\overset{⃑}{F}}_{D}=m_{p}\frac{18\mu }{\rho_{p}d_{p}^{2}}\frac{C_{D}{Re}_{p}}{24}\left(u_{i}-u_{pi}\right) \end{array}$$

${\overset{⃑}{F}}_{R}$ represents the buoyancy force, and its expression is:

(11) $$\begin{array}{c} {\overset{⃑}{F}}_{R}=m_{p}\overset{⃑}{g}\left(1-\frac{\rho }{\rho_{p}}\right) \end{array}$$

${Re}_{p}$ is the Reynolds number for particles, and its expression is:

(12) $$\begin{array}{c} {Re}_{p}=\frac{\rho d_{p}\left|u_{pi}-u_{i}\right|}{\mu } \end{array}$$

$C_{D}$ is the drag coefficient, defined as:

(13) $$\begin{array}{c} C_{D}=\frac{24}{{Re}_{p}}(1+0.15{Re}_{p}^{0.687}) \end{array}$$

where $m_{p}$ is the mass of the particle, $u_{pi}$ represents the particle velocity in the i-direction, μ is the dynamic viscosity of the fluid, $\rho_{p}$ is the density of the particle, $d_{p}$ is the diameter of the particle, and $\overset{⃑}{g}$ is the gravitational acceleration vector.

RSM is a multivariate optimization tool that quantifies the interactive effects of input factors on system responses through quadratic regression modeling. We adopt RSM—a robust design-of-experiments framework—to model nonlinear interactions. In this study, BBD is adopted for experimental design. The advantage of BBD relies on its ability to preserve the precision of regression models with fewer experimental runs. A multifactorial analysis was performed to assess how particle diameter (1–10 μm), peak flow rate (30–90 L/min), and breath-holding (0–8 s) govern drug deposition. Triplicate simulations were run per parameter combination, with outcomes averaged to mitigate random fluctuations. Table 1 lists the factors and levels investigated in the current study (A represents particle diameter, B is short for peak flow rate, C is breath-holding time).

## 3. Results

### 3.1. Model Validation

**Figure 6:**
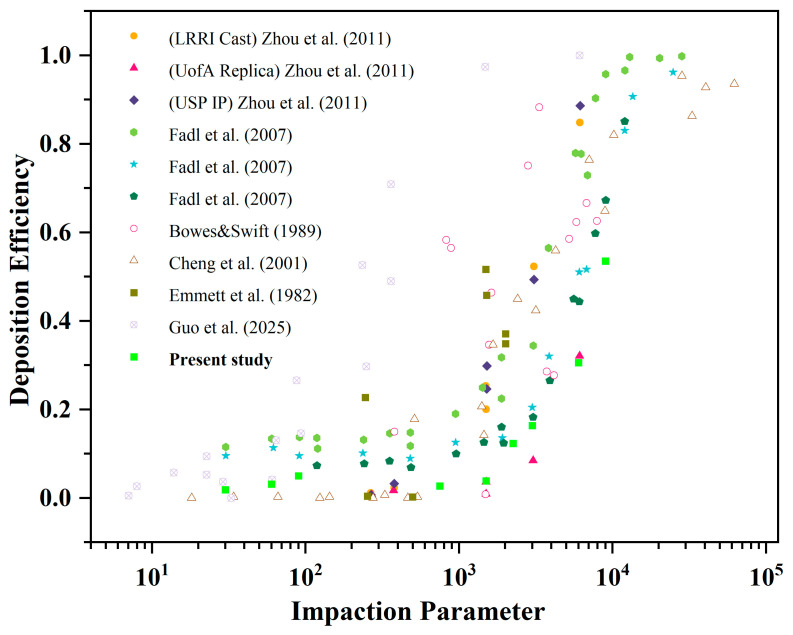
Comparison of particle depositions presented in other literature regardless of the numerical and experimental data [20,27,28,29,30,31].

In the present study, the accuracy and practicality of the numerical simulation were verified as shown in previous work. Given the synthetical effort of the settings of particle diameter and flow rate among different studies, an impaction parameter (IP) should be defined, i.e.:

(14) $$\begin{array}{c} IP=d_{p}^{2}\varrho \end{array}$$

where $d_{p}$ is the particle diameter and Q represents the flow rate. This IP provides a uniform comparison criterion with various particle diameters and flow conditions, regardless of simulation results and clinical trial results. The results of our proposed method are also compared with those from other literature (Figure 6). Although slight numerical discrepancies were observed—attributed to the differences in airway geometric model or numerical simulation methods—the trends in particle deposition efficiency across all models exhibited a high degree of consistency. This consistency confirms the validity of the particle deposition results reported in this work.

### 3.2. Distribution Characteristics of Flow Field

**Figure 7:**
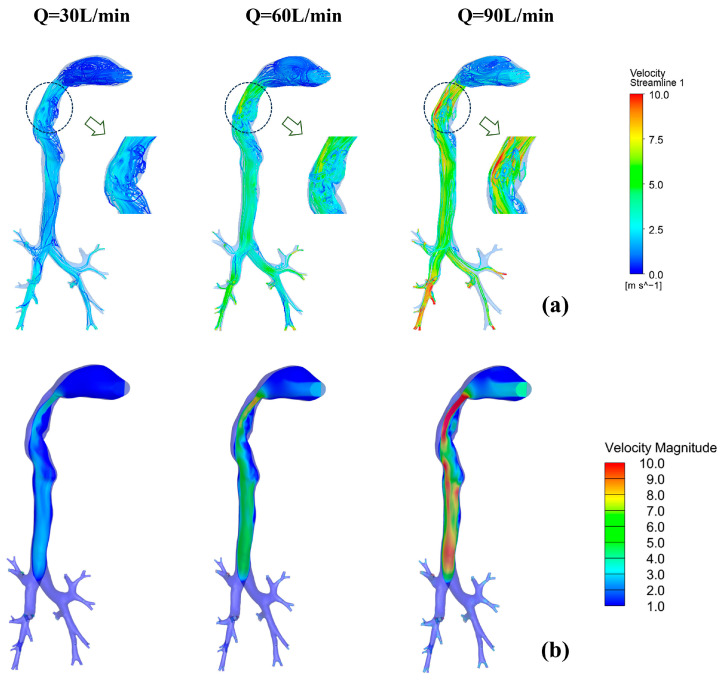
Flow field characteristics at different peak flow rates (Q = 30, 60, 90 L/min). (**a**) Streamline distribution at t = 1.0 s under varying peak flow rates; (**b**) velocity distribution at t = 1.0 s under varying peak flow rates.

During drug inhalation, the complexity of the respiratory tract structure results in variations in the internal flow field. Figure 7 presents the flow field characteristics at three peak flow rates (30, 60, and 90 L/min). Figure 7a,b demonstrate that while flow patterns remain analogous across varying peak flow rates in the airway, the pharyngeal turbulence intensity is markedly amplified with elevated inlet flow rates. As can be seen from the figure, the high-velocity regions of gases under different peak flow rates are primarily concentrated in the pharyngeal region. This is due to the abrupt reduction in the cross-sectional area of the airway, which causes a sharp increase in flow velocity. Combined with the uneven velocity distribution induced by the complex anatomical structures in this region, turbulence forms here, and its intensity intensifies with increasing peak flow rates. Additionally, gas flows under varying peak velocities generate a pharyngeal “jet flow,” which redirects the high-speed region toward the posterior trachea. Following this redirection, the velocity profile progressively stabilizes and becomes uniform along the tracheal lumen.

**Figure 8:**
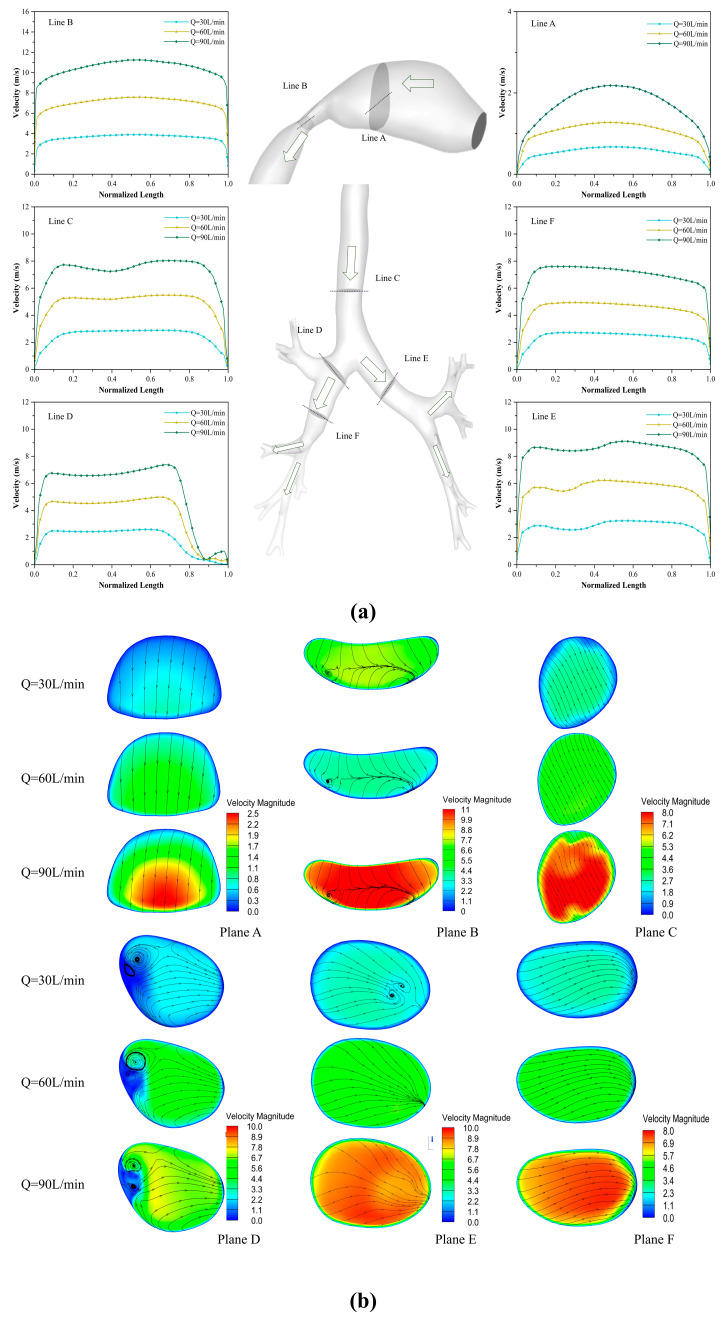
(**a**) Normalized axial velocity distribution along different cross-sectional axes at t = 1 s under peak flow rates of 30, 60, and 90 L/min. (**b**) Instantaneous streamline diagrams of different cross-sections at 1.0 s under peak flow rates of 30, 60, and 90 L/min.

To further investigate how cross-sectional area changes affect fluid dynamics, we analyzed instantaneous velocity contours along axial cross-sections and normalized velocity profiles at t = 1.0 s under varying positions and peak flow rates (Figure 8). The results demonstrate that the cross-sectional area governs the flow behavior within the model.

Due to the larger cross-sectional area of the oral cavity, the flow velocity at cross-section A is consistently lower than the inlet velocity. In contrast, cross-section B is located at the pharynx and larynx, where the reduced cross-sectional area leads to a sharp increase in flow velocity. Consequently, the flow velocities at cross-section B are uniformly higher than the inlet velocity. Vortex structures are evident on the D- and E-planes. With rising flow velocity, vortices on the D-plane exhibit progressive size reduction, whereas those on the E-plane are fully suppressed. These observations confirm that flow velocity critically governs both the spatial distribution and scaling of vortical features.

**Figure 9:**
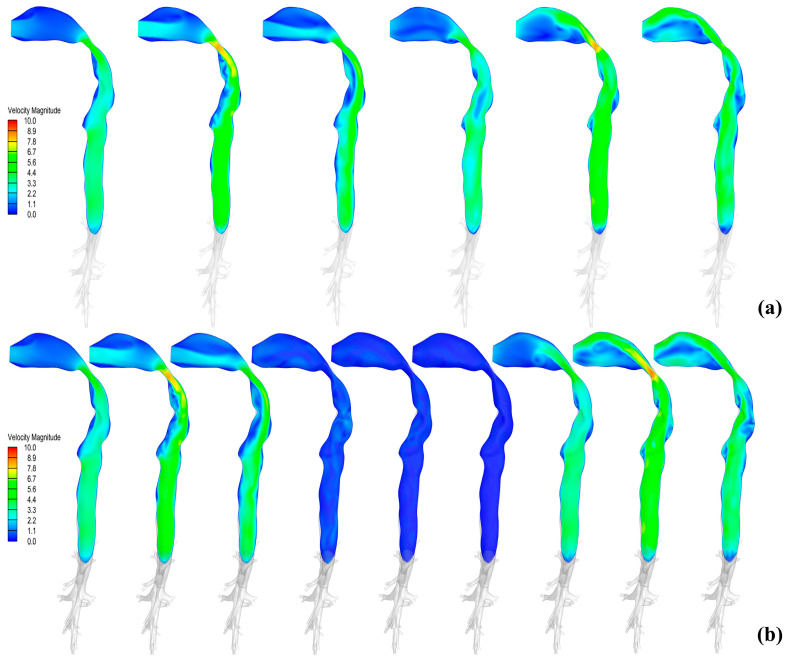
(**a**) Instantaneous velocity contour plots at a peak flow rate of 60 L/min per 0.5 s (a) without breath-holding; (**b**) with breath-holding (2.0 s).

Figure 9 provides a detailed analysis of the transient flow field dynamics of drug inhalation. Figure 9a captures velocity fluctuations across the inhalation–exhalation sequence, whereas Figure 9b reveals how velocity profiles evolve during a 2 s breath hold. In Figure 9b, during the inhalation phase, the velocity distribution aligns with the cyclic breathing pattern observed in Figure 9a. The breath-holding phase induces dynamic changes due to airflow interruption: weak gas recirculation is detected in the trachea at 0.5 s, while airflow velocity diminishes to near-zero levels after 1.0 s. During the exhalation phase, the flow characteristics revert to the cyclic breathing pattern, consistent with Figure 9a.

### 3.3. Pattern of Particle Distribution

For optimal therapeutic impact, drugs must be delivered precisely to target regions. To reduce transport losses and systematically evaluate airway deposition patterns, we introduce wall deposition fraction (DF) and deposition efficiency (DE), which quantitatively characterize the spatial distribution and retention of particles within the respiratory tract model.

The DF is defined as:

$$\frac{number\;of\;deposited\;particles\;on\;a\;specific\;wall\;section}{total\;number\;of\;deposited\;particles\;on\;the\;respiratory\;tract\;wall}\times 100\%$$

The DE is defined as:

$$\frac{total\;number\;of\;particles\;deposited\;on\;the\;wall+total\;number\;of\;particles\;escaping\;through\;the\;outlet}{total\;number\;of\;particles\;entering\;the\;respiratory\;tract}\times 100\%$$

**Figure 10:**
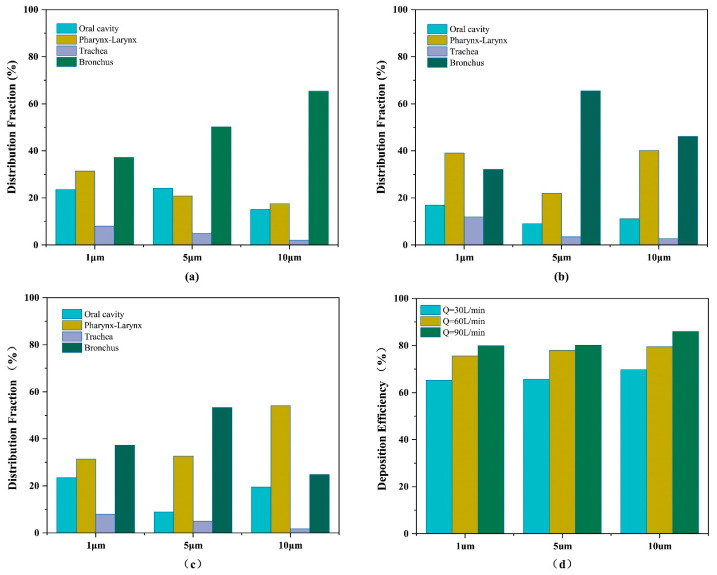
Distribution fractions of different-sized particles in various locations. The peak flow rates are (**a**) Q = 30 L/min, (**b**) Q = 60 L/min, and (**c**) Q = 90 L/min. (**d**) Particle deposition efficiency at peak flow rates of 30, 60, and 90 L/min and particle diameters of 1, 5, and 10 μm.

First, we investigate the interplay between peak flow rate and particle diameter within dynamic respiratory cycles. Figure 10 displays the DF and DE of particles of different diameters (1, 5, 10 μm) under various conditions of inlet peak flow rate (30, 60, and 90 L/min).

Figure 10d reveals that DE scales positively with particle diameter and peak flow rate. For a fixed Q, DE rises with d due to enhanced inertial impaction. For a constant d, higher Q amplifies turbulent mixing, further elevating DE. At a low peak flow rate (30 L/min), Figure 10a reveals predominant particle deposition in the bronchial region. Laminar flow dominance suppresses inertial impaction, favoring advection-driven particle transport to distal airways. Particle accumulation in the oral–pharyngeal region stems from the abrupt narrowing of the airway cross-section. This constriction elevates airflow velocity, amplifying inertial forces and driving particle deposition via impaction. The trachea’s streamlined geometry minimizes flow disturbances, reducing turbulent deposition. Consequently, particle retention in this region is significantly lower.

At 60 L/min, Figure 10b demonstrates a 27.82–62.48% reduction in oral particle deposition compared to 30 L/min. This stems from increased airflow inertia, which suppresses local deposition and enhances distal transport. At 90 L/min, Figure 10c reveals amplified jet flows in the pharyngeal constriction, driving particle impaction. This results in a rise in pharyngeal deposition compared to lower peak flow rates.

Building upon these findings, we further incorporate breath-holding into a multifactorial analytical framework to investigate its impact on particle deposition within dynamic respiratory cycles while simultaneously dissecting the interaction mechanisms between breath-holding time and the aforementioned two factors. The experiment design based on BBD is shown in Table 2. Thirteen cases with various parameters (the particle sizes ranged from 1 μm to 10 μm, the breath-holding time from 0 s to 8 s, and the inlet peak flow rate from 30 L/min to 90 L/min) were simulated, and their outcomes were used to train the RSM.

With DE defined as the response variable (Y), the simulation data were analyzed and trained into an RSM with the SAS RSREG ® 9.4M6 (TS1M6) (SAS Institute Inc., 2020, Cary, NC, USA), and its performance was evaluated by the coefficient of determination ($R^{2}$) and analysis of variance (ANOVA). The quadratic polynomial regression equation is Y = 84.55 + 5.95A + 5.13B + 5.53C − 3.74$C^{2}$ (where $R^{2}$ = 0.9902), and the details of the variance analysis are shown in Table 3.

**Figure 11:**
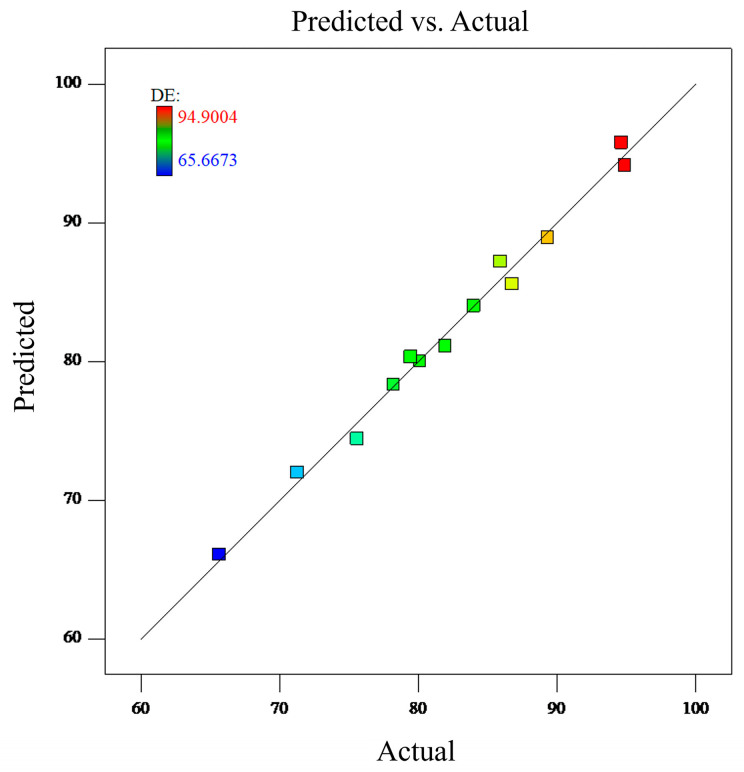
Comparison between the particle deposition efficiency predicted by the regression model and the actual results from CFD simulations.

$R^{2}$ = 0.9902 confirms that 99.02% of the response variance is attributable to the chosen variables, which validates the model’s explanatory power. The *p*-value of 0.0074 is significantly lower than 0.05 (*p* < 0.05), which demonstrates that the fitted model is statistically significant. In other words, it can be concluded that the trained RSM has high accuracy in directly predicting the particle deposition efficiency with various parameters. The particle deposition efficiency predicted by the regression model and the results from CFD simulations are shown in Figure 11.

Further analysis revealed that breath-holding exerts a statistically significant impact on particle deposition efficiency. Among the investigated factors, peak flow rate dominates the modulation of DE, followed by breath-holding duration and particle diameter. In terms of interaction effects, the significance of the hierarchy is BC > AB > AC.

The regression equation explicitly defines the functional relationships between the three factors (peak flow rate, breath-holding duration, and particle diameter) and DE. This data-driven framework provides actionable insights for optimizing inhalation therapy protocols.

**Figure 12:**
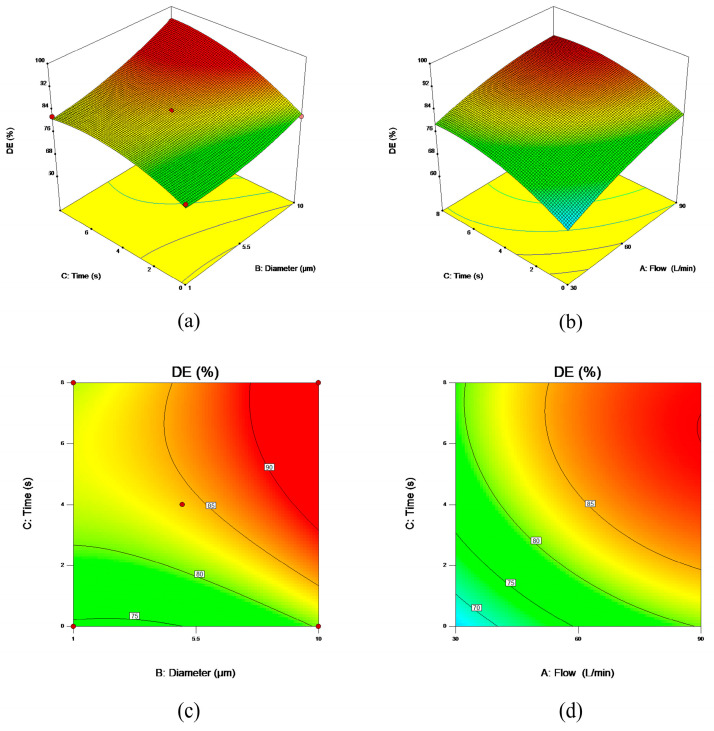
Three-dimensional response surface analysis and two-dimensional contour analysis between parameters. (**a**) 3D response surface: interaction between particle diameter and breath-holding time; (**b**) 3D response surface: interaction between peak flow rate and breath-holding time; (**c**) 2D contour plots: interaction between particle diameter and breath-holding time; (**d**) 2D contour plots: interaction between peak flow rate and breath-holding time.

Three-dimensional (3D) response surface plots and two-dimensional (2D) contour plots were generated (Figure 12). As shown in Figure 12, prolonging breath-holding time significantly enhances particle deposition efficiency. As shown in Figure 12a, under a constant peak flow rate, particles of different diameters exhibit a 5.8078–15.1732% increase in DE after an 8 s breath hold compared to no breath-holding. Additionally, larger particles demonstrate a more rapid short-term enhancement in DE during breath-holding, driven by inertial impaction mechanisms. As shown in Figure 12b, when the particle diameter is held constant, DE increases by 9.4015–12.3142% after an 8 s breath hold compared to no breath-holding across different peak flow rates. Notably, lower peak flow rates exhibit greater sensitivity to breath-holding time, with DE enhancement being more pronounced under such conditions.

## 4. Discussion

Inhalation drug delivery faces challenges in particle visualization and tracing, complicating carrier quantification and localization. Systematic elucidation of deposition mechanisms is crucial for guiding delivery [20].

The results of this study indicate that, under high flow rate conditions, enhanced airflow inertia leads to inertial impaction, resulting in the predominant deposition of large-diameter particles in the upper airways. In contrast, reduced inertial effects at low flow rates allow particles to penetrate deeper into the lower respiratory tract. Gravitational sedimentation becomes the dominant mechanism, particularly during breath-holding, when airflow velocity diminishes to near-zero levels. Additionally, small particles are also influenced by Brownian diffusion under low flow rates and during breath-holding, resulting in an increase in deposition with prolonged residence time. In the administration of respiratory drugs, such as for the treatment of chronic obstructive pulmonary disease (COPD), it is important to minimize drug residence in the oral and pharyngeal areas and promote drug deposition deeper into the bronchi to enhance drug utilization. As shown in Figure 10d, the DE at a peak flow rate of 60 L/min is 9.78–12.29% higher than that at 30 L/min, and the DE at 90 L/min is 2.16–6.49% higher than that at 60 L/min. However, at a peak flow rate of 60 L/min, the DF of particles with sizes 5 and 10 μm in the bronchi is 12.17% and 21.25% higher, respectively, than that at a peak flow rate of 90 L/min. These data indicate that a peak flow rate of 60 L/min is more conducive to the deep deposition of particles. At a peak flow rate of 60 L/min, the DE of particles of size 5 μm differs by 1.51% from that of 10 μm-sized particles and by 2.37% from that of 1 μm-sized particles. However, as shown in Figure 10b, the DF of 5 μm-sized particles in the bronchi is 19.46% higher than that of 10 μm-sized particles and 33.44% higher than that of 1 μm-sized particles. Considering these factors, a particle diameter of ~5 μm and a peak flow rate of 60 L/min are more conducive to the deep deposition of drugs. These conclusions are consistent with previous research findings [32,33].

After drug inhalation into the respiratory tract, a significant portion of the medication remains suspended in the oral cavity and airways at the end of the inhalation phase. If exhalation occurs immediately, many suspended particles are expelled with the exhaled airflow, thereby reducing drug deposition efficiency. Prolonged breath-holding facilitates particle deposition by allowing sufficient time for gravitational settling and Brownian diffusion to enhance retention in target regions.

## 5. Conclusions

During the process of drug inhalation, the deposition effect is primarily influenced by airway morphology, respiratory pattern, and particle properties. In this study, a high-fidelity three-dimensional airway model was established, spanning from the oral cavity to the fifth-level bronchus, revealing that the cross-sectional area of the airways is a critical factor influencing flow field distribution. Peak flow rates of 30, 60, and 90 L/min were selected to simulate different breathing intensities. Particle diameters of 1, 5, and 10 μm were chosen to represent different drug diameters. Breath-holding times of 2, 3, 4, 6, and 8 s were selected to represent various breath-holding times. Our data reveal that an increase in both the peak flow rate and particle diameter is positively correlated with the deposition efficiency of particles in the respiratory tract. Specifically, under low-flow conditions, small-diameter particles require longer breath-holding times to achieve sufficient deposition.

Our research data contribute to further refining the administration strategies for inhaled medications. Taking a patient with COPD as an example, with pulmonary dysfunction and low inhaled flow rate, the breath-holding time should last much longer, or the device should be designed with a larger dosage or flow rate.

Despite the verification of the particle depositions with the previous literature, our findings will be further validated by some in vitro experiments based on a certain platform of electronic lung simulation devices. Also, we will undertake targeted exploration through the development of multiphysics-coupled pathological airway models to dissect the disease-specific particle deposition mechanism.

## Figures and Tables

**Table 1:** Setting of factor levels and their symbols used in RSM. The particle sizes range was selected from 1 μm to 10 μm, the breath-holding time was selected from 0 s to 8 s, and the inlet peak flow rate was selected from 30 L/min to 90 L/min.

level	(A) Peak Flow Rate (L/min)	(B) Particle Diameter (μm)	(C) Breath-Holding Time (s)
−1	30	1	0
0	60	5	4
1	90	10	8

**Table 2:** Experiment design based on BBD. Thirteen cases were simulated and used for RSM training. The particle sizes ranged from 1 μm to 10 μm, the breath-holding time from 0 s to 8 s, and the inlet peak rate from 30 L/min to 90 L/min.

Case No.	A(L/min)	B(μm)	C(s)	DE(%)
1	90	5	0	80.1205
2	90	1	4	85.9185
3	60	1	8	81.9384
4	60	5	4	84.0030
5	30	5	0	65.6673
6	90	5	8	89.3506
7	60	1	0	75.5906
8	30	10	4	86.7918
9	60	10	0	79.4711
10	30	5	8	78.2122
11	60	10	8	94.6443
12	30	1	4	71.3084
13	30	10	4	94.9004

**Table 3:** Variance analysis of response surface methodology model.

Source	Sum of Squares	Df	Mean Square	F Value	Pr > F
Model	986.28	9	95.59	33.64	0.0074
A-flow	281.19	1	281.19	98.97	0.0022
B-diameter	210.66	1	210.66	74.14	0.0033
C-time	243.47	1	243.47	85.69	0.0027
AB	11.02	1	11.02	3.88	0.1435
AC	2.75	1	2.75	0.97	0.3980
BC	19.29	1	19.29	6.79	0.0800
A^2^	8.46	1	8.46	2.98	0.1830
B^2^	9.85	1	9.85	3.47	0.1595
C^2^	32.01	1	32.01	11.27	0.0439
Residual	8.52	3	2.84		
Cor total	868.80	12			

## Data Availability

The original contributions presented in this study are included in the article. Further inquiries can be directed to the corresponding author.

## Abbreviations

The following abbreviations are used in this manuscript:

CFD	Computational Fluid Dynamics
COPD	Chronic Obstructive Pulmonary Disease
CT	Computed Tomography
DPM	Discrete Phase Model
RSM	Response Surface Methodology
BBD	Box-Behnken Design
DF	Deposition Fraction
DE	Deposition Efficiency
2D	Two-dimensional
3D	Three-dimensional
